# Traumatic Brain Injury-Induced Dysregulation of the Circadian Clock

**DOI:** 10.1371/journal.pone.0046204

**Published:** 2012-10-03

**Authors:** Deborah R. Boone, Stacy L. Sell, Maria-Adelaide Micci, Jeanna M. Crookshanks, Margaret Parsley, Tatsuo Uchida, Donald S. Prough, Douglas S. DeWitt, Helen L. Hellmich

**Affiliations:** Department of Anesthesiology, University of Texas Medical Branch, Galveston, Texas, United States of America; Florida State University, United States of America

## Abstract

Circadian rhythm disturbances are frequently reported in patients recovering from traumatic brain injury (TBI). Since circadian clock output is mediated by some of the same molecular signaling cascades that regulate memory formation (cAMP/MAPK/CREB), cognitive problems reported by TBI survivors may be related to injury-induced dysregulation of the circadian clock. In laboratory animals, aberrant circadian rhythms in the hippocampus have been linked to cognitive and memory dysfunction. Here, we addressed the hypothesis that circadian rhythm disruption after TBI is mediated by changes in expression of clock genes in the suprachiasmatic nuclei (SCN) and hippocampus. After fluid-percussion TBI or sham surgery, male Sprague-Dawley rats were euthanized at 4 h intervals, over a 48 h period for tissue collection. Expression of circadian clock genes was measured using quantitative real-time PCR in the SCN and hippocampus obtained by laser capture and manual microdissection respectively. Immunofluorescence and Western blot analysis were used to correlate TBI-induced changes in circadian gene expression with changes in protein expression. In separate groups of rats, locomotor activity was monitored for 48 h. TBI altered circadian gene expression patterns in both the SCN and the hippocampus. Dysregulated expression of key circadian clock genes, such as *Bmal1* and *Cry1,* was detected, suggesting perturbation of transcriptional-translational feedback loops that are central to circadian timing. In fact, disruption of circadian locomotor activity rhythms in injured animals occurred concurrently. These results provide an explanation for how TBI causes disruption of circadian rhythms as well as a rationale for the consideration of drugs with chronobiotic properties as part of a treatment strategy for TBI.

## Introduction

In humans, circadian disturbances have been linked to many physiological and psychological consequences such as cancer, diabetes, metabolic disorders, hypertension, depression, bipolar disorder, and learning and memory dysfunction [1], [2]. Disruption of circadian rhythms may also contribute to several of the pathophysiological consequences of traumatic brain injury (TBI). Reports of circadian dysregulation in TBI patients include altered homeostatic mechanisms such as regulation of blood pressure, heart rate, body temperature [3], and hormone cycles [4], as well as the sleep-wake cycle [5], [6]. Moreover, circadian disturbances can inhibit neurogenesis, which is a critical component of recovery after TBI [7].

It is well established that patients recovering from TBI are prone to sleep-wake cycle disturbances, which suggests dysregulation of the central timing mechanism that regulates sleep [8]–[10]. The suprachiasmatic nuclei (SCN), located in the anterior hypothalamus, are the anatomical location of the endogenous master clock that controls circadian rhythms. The SCN communicate with peripheral “clocks” to regulate homeostasis and other cyclic physiological patterns (see Golombek & Rosenstein, 2010 for review) [11]. The internal circadian rhythm generated by the SCN is controlled by a cycle of gene expression changes in SCN pacemaker neurons that entrain physiological functions and behavior to external stimuli for proper adaptation to daily temporal cycles in the environment. Signals from the SCN are communicated throughout the brain and the body to various peripheral clocks. One of the important recipients of circadian signaling in the brain is the hippocampus, which is vital for cognitive functions such as learning and memory and is particularly vulnerable to TBI [12], [13].

Circadian clock genes are expressed in many brain regions outside the SCN, including the hippocampus [14], and circadian rhythms have long been known to regulate hippocampal function [15]. There is strong evidence that the endogenous circadian clock modulates long-term potentiation (LTP) – an electrophysiological indication of synaptic plasticity – in the hippocampus [16]. In fact, an operational circadian system appears to be crucial for hippocampal-dependent learning to function properly [17]. For example, in rats, chronic phase shifting of the light-dark cycle impaired both acquisition and retention of platform location in a hippocampal-dependent water maze task [18]. Thus, we speculated that injury-induced alterations in circadian clock genes might contribute to learning and memory dysfunction often reported in human TBI patients [19]. The association between circadian dysfunction and learning and memory problems may be the result of disrupting sensitive time-dependent neural circuitry and/or may be mediated by molecular substrates such as the cyclic-adenosine monophosphate (cAMP)/mitogen-activated protein kinase (MAPK)/cAMP response element binding protein (CREB) signal transduction pathways that are common between the SCN and the hippocampus and many other cells and tissues [20], [21].

In the SCN, protein products of the circadian locomotor output cycles kaput (*Clock*) gene and the brain and muscle aryl hydrocarbon receptor nuclear translocator (ARNT)-like *(Bmal1)* gene heterodimerize in the nucleus and induce target genes including Period (isomers *Per1* and *Per2*) and Cryptochrome (isomers *Cry1* and *Cry2*), that encode proteins, which in turn, heterodimerize in the cytoplasm, translocate to the nucleus and inhibit CLOCK:BMAL1-mediated transcription [22]. This transcriptional-translational feedback loop functions in an autoregulatory (activation/repression) cyclic fashion. For example, SCN rhythms can be regulated by MAPK activity through decreasing basal *Bmal1* expression levels and thereby decrease firing rhythms [23], [24]. This is a process in common with time-of-day expression and persistence of hippocampal-dependent memory, which depends on functioning SCN [25]. These aforementioned processes signify conservation of cAMP/MAPK/CREB-dependent mechanisms between anatomical locations that underlie neuronal plasticity mechanisms for learning and memory.

Thus, using naïve rats and rats subjected to moderate fluid-percussion TBI and sham-injury, we addressed the hypothesis that TBI disrupts the cyclic pattern of circadian clock and clock-associated gene expression in the SCN as well as in the hippocampus (which is particularly vulnerable to injury). We also investigated whether TBI altered the expression of injury-associated genes (such as brain-derived neurotrophic factor [BDNF]) in these two brain areas. Specific brain areas were isolated by laser capture microdissection (LCM) or manual microdissection, and gene expression was quantified by real-time PCR. Confirmation of changes in gene expression was assessed using analysis of protein products of selected genes of interest. Since daily rhythms of rodent locomotor activity are well known to be controlled by the master pacemaker in the SCN, we measured locomotor activity as a functional outcome [26]. This is the first investigation of the effects of TBI on circadian clock gene expression in the SCN and the hippocampus.

## Materials and Methods

### Animals

Adult male Sprague-Dawley rats (350 g–400 g) from vendor Charles Rivers (Portland, Maine) were housed 2 per cage with food and water ad libitum in a vivarium with these constant conditions: light cycle (6∶00–18∶00) temperature (21°C–23°C), and humidity (40%–50%). All animal experiments were approved by the Institutional Animal Care and Use Committee of the University of Texas Medical Branch, Galveston, Texas and conducted according to the National Institutes of Health Guide for the Care and Use of Laboratory Animals (8^th^ edition, National Research Council).

### Surgical Preparation and Lateral Fluid-Percussion TBI

To standardize the time of injury, moderate lateral fluid percussion TBI or sham injury was performed at the same time (12∶00) each day. Male Sprague-Dawley rats weighing 350 g to 400 g were anesthetized with 4% isoflurane, intubated, and mechanically ventilated (NEMI Scientific; New England Medical Instruments, Medway, MA) with 1.5% to 2.0% isoflurane in oxygen:air (70∶30). Rectal and temporalis muscle temperatures were monitored using a Physitemp Thermalert Model TH-8 (Physitemp Instruments, Inc., Clifton, NJ), and rectal temperature maintained at 37°C throughout the procedure, using a thermostatically controlled water blanket (Gaymar Industries, Inc., Orchard Park, NY).

Rats were prepared for lateral fluid-percussion TBI according to the procedure developed and characterized by McIntosh, et al. (1989) [27]–[29]. Fluid-percussion TBI is a well-established model of closed-head injury that reproduces aspects of human TBI, including cerebrovascular [30] and pathophysiological, neurological and behavioral responses [31]. Rats were placed in a stereotaxic head holder, a midline incision was made in the scalp, and the fascia was removed to expose the skull. Using a Michele trephine, a parasagittal craniotomy was performed 3 mm to the right of the sagittal suture, midway between the bregma and lambda sutures. The bone chip was removed exposing the intact dura. A modified 20-gauge needle hub was secured in the craniotomy site, and cemented in place with hygienic dental acrylic. Once the acrylic was solidified, isoflurane was discontinued and preparation was made for the delivery of the fluid pulse. The trauma device was connected to the rat by a tube that terminated with a male adapter that connected to the modified needle hub. The device consists of a Plexiglas cylinder (60 cm long and 4.5 cm in diameter) filled with isotonic saline connected to a hollow metal cylinder housing a pressure transducer (Statham PA856-100; Data Instruments, Acton, MA, USA) and closed by a Plexiglas piston mounted on O-rings. After the return of a withdrawal reflex to a paw pinch the rats were subjected to a moderate (2.0 atm) pressure pulse. The pulse was delivered by a 4.8 kg steel pendulum striking the piston after being dropped from the appropriate height (determined by prior optimization using an oscilloscope to record the pressure). After TBI or sham injury (no pressure pulse delivered), rats were disconnected from the device and righting reflex was assessed every 60 seconds until a normal response was observed. Rats were then placed on 2% isoflurane, and wound sites were infused with bupivacaine and sutured. Isoflurane was discontinued and rats were extubated and allowed to recover in a warm humidified chamber.

### Experimental Design

#### Experiment 1: Circadian clock gene expression in the rat SCN and hippocampus after TBI

After fluid-percussion TBI or sham surgery, rats were euthanized at 4 h intervals, over a 48 h period (n = 6 rats per experimental group/time point). Injured and sham-injured rats were anesthetized with isoflurane for surgical procedures. Since anesthetics perturb circadian rhythms and can impair cognition in postoperative patients [32], [33], tissue was collected at 4 h intervals over a 24 h period from naïve rats to assess the effects of anesthetics. The ipsilateral hippocampus was removed and placed in *RNAlater* (Ambion, now Life Technologies, Grand Island, NY) and stored at 4°C; the remaining brain was fresh frozen on dry ice for LCM and stored at −80°C.

For total RNA Isolation from the hippocampus, each hippocampus was homogenized in 1 ml of UltraSpec (Biotecx, Houston, TX) and total RNA was isolated using the manufacturer’s protocols. RNA samples in 15 µl to 20 µl of nuclease-free water were DNase treated using the Turbo-DNase kit (Ambion) at 37°C for 30 min. Total RNA was quantified on a Nano-drop spectrophotometer (Thermo-scientific, Wilmington, DE).

LCM of suprachiasmatic nuclei was performed using 10 µm coronal sections cut on a cryostat and mounted on uncoated, pre-cleaned, superfrost glass slides (Fisher Scientific, Pittsburgh, PA). When the SCN region of the brain was reached (located above the optic chiasm), every section was collected through the optic chiasm. The frozen sections were thawed at room temperature for 30 seconds and fixed in 75% ETOH for 1 min. After fixation, the slides were briefly rinsed in RNase-free water (1 min), stained with 1% cresyl violet (1 min), rinsed in RNase-free water, dehydrated in 95% ethanol, 100% ethanol and cleared in xylene. The sections were then air-dried for 10 to 15 min in a fume hood. All solutions were prepared with RNase-free water, and the cresyl violet was sterile filtered with a 0.22 µM filter. Each section was viewed under the microscope to confirm the presence of the SCN. LCM was performed using a PixCell IIe laser capture microscope with an infrared diode laser (Life Technologies). Both lobes of the SCN were captured on the thermoplastic film of a CapSure macro LCM cap (Life Technologies). The smallest laser spot size (7.5 µm) was used with a power setting of 75 mW to 100 mW and pulse duration of 0.85 ms to 1.5 ms, for optimum capture of the cells. The CapSure caps that contained the SCN neurons were transferred to 0.5 ml tubes filled with lysis buffer solution from the RNAqueous-Micro kit (Ambion). Samples were vortexed to insure cell lysis and stored at −80°C until RNA isolation.

Total RNA was isolated from the LCM SCN neurons using the RNAqueous-Micro kit (Ambion) according to the manufacturer's protocols. Total RNA was eluted in 20 µl of nuclease-free water and DNase1 treated at 37°C for 20 min to remove trace amounts of genomic DNA.

The total RNA from the SCN neurons was linearly amplified using the Message Amp II aRNA kit (Ambion) according to the manufacturer’s protocol. The amplified RNA was quantified on a Nanodrop spectrophotometer (Thermoscientific).

Total hippocampal RNA (500 ng) and SCN RNA (80 ng) was reverse transcribed as previously described [34]. The reactions were incubated for 10 min at 25°C, then 30 min at 48°C, and 5 min at 95°C in a thermocycler (TechGene), centrifuged and then stored at −20°C until use. Quantitative real-time PCR was performed on a MX3000P multiplex PCR system (Stratagene, La Jolla, CA) with Taqman reagents (Applied Biosystems, now Life Technologies, Grand Island, NY) as previously described [34]. The level of each gene was normalized to GAPDH and analyzed by the MXPro software (Stratagene). Values were expressed in quantities relative to the calibrator. Relative quantification can be used to determine fold increases and decreases in gene expression compared with a certain sample or “calibrator.” The calibrator was run on each PCR plate through the entire experiment. For these experiments, whole rat brain RNA was used as the calibrator. Probe and primer sequences of clock genes, clock-associated genes and injury-induced genes are shown in Table S1.

#### Experiment 2: Confirmation of circadian clock gene expression changes in the hippocampus by protein analysis

To confirm that gene expression data correlated with changes in protein levels, a time point was chosen where the expression of the circadian clock gene was different in tissue from injured rats compared with that of naïve or sham-injured rats. For these experiments, rats were subjected to moderate fluid-percussion TBI or sham injury as described above. In addition, naïve rats were included and all tissue was collected at the same time-point post injury, or time of day in the case of the naïve rats.

For immunohistochemical analysis, rats were perfused with 4% paraformaldehyde and sacrificed 20 h after TBI at 08∶00. Naïve rats were also sacrificed at 08∶00. Each brain was dissected and post-fixed in paraformaldehyde, rinsed in PBS, embedded in 30% sucrose and stored at 4°C until sectioned. Brains were embedded in Tissue-Tek O.C.T. (Sakura, Hayward, CA) for sectioning. Coronal sections (10 µm) of the hippocampus were mounted on pre-cleaned, plus slides (VWR, West Chester, PA). Immunofluorescence localization was performed as follows: sections were hydrated in PBS at room temperature and blocked in 5% normal goat serum/0.3% Triton X-100 in PBS. Slides were then incubated with a primary antibody diluted in 1.5% normal goat serum/0.3% Triton X-100 in PBS overnight at 4°C. Sections were washed in PBS and incubated in an ALEXA-conjugated antibody diluted in 1.5% normal goat serum/0.3% Triton X-100 in PBS at room temperature in the dark. Then, sections were washed in PBS, rinsed in dH_2_0, mounted in hard set-DAPI (Vecta-Shield, Vector laboratories, Inc. Burlingame, CA) and cover slipped. All antibodies were purchased from Santa Cruz Biotechnology Inc. (Per2 # H-90, Cry1 # H-84) except for *Bmal1* (catalog # Ab3443, Abcam, Cambridge, MA). Mean fluorescence intensity levels were calculated for each image and corrected for the background using ImageJ software (NIH).

For Western blot analysis, rats were subjected to moderate TBI or sham injury as described above. Naïve rats were also included. Rats were sacrificed 20 h after moderate TBI or sham injury at 08∶00 and 32 h after injury at 20∶00 h. Hippocampi were dissected and frozen on dry ice. Tissue was homogenized in an ice-cold buffer containing 2% sodium dodecyl sulphate (SDS), protease cocktail inhibitor (Sigma-Aldrich), 1 mM phenylmethylsulphonyl fluoride (PMSF), 1 mM dithiothreitol (DTT), 5 mM ethylenediaminetetraacetic acid (EDTA) in 50 mM Tris-HCl, pH 7.4, incubated on ice for 5 min and then centrifuged at 14,000 g for 10 min at 4°C. Protein concentrations were determined by means of the bicinchoninic acid (BCA) assay (Thermo Scientific, Rockford IL) using a Nano-drop spectrophotometer (Thermo Scientific, Rockford IL) according to the manufacturer’s instructions. Proteins (40 µg) were separated on an SDS-PAGE 8% to 16% gel and transferred to 0.2 µm PVDF membranes. Blots were blocked using a blocking solution (BS: 5% non-fat dry milk in TBS containing 0.1% Tween 20 (TBS-T), then incubated with a mouse anti-Period 2 antibody (catalog # 611138, BD Biosciences, San Jose, CA) diluted 1∶1000 in BS at 4°C overnight. Blots were then incubated with an anti-mouse HRP conjugated secondary antibody (catalog # 7076, Cell Signaling, Danvers, MA) diluted 1∶3000 in BS for 1 h at room temperature. The blots were washed in TBS-T, followed by incubation for 5 min in enhanced chemiluminescent solution (ECL plus, GE Healthcare), and exposed onto X-ray films for varying lengths of time (5 sec to 1 min). To normalize the expression of Period 2, the blots were re-incubated with a mouse anti-GAPDH antibody (catalog # ab8245, Abcam, Cambridge, MA) diluted 1∶10,000 in BS. Blots were incubated with an anti-mouse HRP-conjugated secondary antibody (catalog # 7076, Cell Signaling, Danvers, MA) diluted 1∶3000 in BS. The intensities of the bands were quantified using UN-SCAN-IT software (Silk Scientific Corporation, Orem, UT) and PER2 protein expression was normalized to the expression of the housekeeping gene GAPDH (n = 3).

#### Experiment 3: Circadian locomotor activity after TBI

Rats were either naïve or received a sham injury or moderate TBI as described above (n = 6). Surgical procedures were performed at 12∶00 each day; 2 h after recovery from anesthesia, rats were placed in the activity-monitoring home cages with food and water *ad libitum*. Locomotor behavior was monitored after injury using the Cage Rack Photobeam System with Flex-Field software (San Diego Instruments, San Diego, CA), which quantifies rat locomotor activity using a 4 × 8 photo-beam configuration. Beams were set 5 cm above the cage floor to track horizontal activity. The number of beam breaks were counted and categorized as either peripheral or central activity. Data was collected in 30 min bins over a 48 h period.

### Statistical Analysis

Each circadian clock gene was analyzed for expression in the hippocampus and SCN separately. Data analyses were conducted using analysis of variance (ANOVA) for a two-factor experiment. The two factors were treatment group (naïve-day1, sham-day1, sham-day2, TBI-day1, TBI-day2) and time of day (16∶00, 20∶00, 24∶00, 04∶00, 08∶00, and 12∶00). Main effects and interactions were assessed at the α = 0.05 level of significance. Multiple comparisons were conducted using a *t* statistic with the standard error computed from the residual mean square in the ANOVA. Since more than 100 comparisons were tested, the 0.0005 level was used for comparison-wise error rate. Statistical computations were carried out using PROC GLM in SAS®, Release 9.1 [35].

For immunohistochemistry data, fold changes between the mean fluorescence intensities were measured and calculated, using ImageJ software, in sections from TBI and naïve rat brains, and analyzed using the one sample *T*-test.

Western blot data were analyzed for PER 2 protein at each time point using ANOVA for a single-factor experiment, i.e injury group (naïve, sham and TBI). The injury group was assessed at the α = 0.05 level of significance. Multiple comparisons were conducted using Fisher’s least significant difference procedure with Bonferroni adjustment for the number of comparisons. Statistical computations were carried out using PROC GLM in SAS®, Release 9.1 [35].

Our locomotor activity experiment is a two-factor experiment with repeated measures. The two factors are treatment group (naïve, sham, TBI) and time (48 h and 5 periods). Data analysis was conducted using the SAS® system, Release 9.1 [35]. Main effects and interactions were assessed at the α = 0.05 level of significance. Multiple comparisons were conducted using Fisher’s least significant difference procedure with the Bonferroni adjustment for the number of comparisons.

## Results

### TBI Effects on the Expression of Circadian Clock Genes in the SCN and Hippocampus

We first confirmed, by quantitative, real-time PCR analysis, previous reports showing that several circadian-related genes such as *Bmal1*, *Clock*, *Cry1*, and Period 1, 2 and 3 (*Per1, Per2, Per3*) and Timeless (*Tim*), are present in the SCN and the hippocampus of naïve rats and are expressed in an oscillating manner (Figure S1A). Moreover, in addition to the circadian-related genes, we found that injury-induced genes in the hippocampus, including BDNF, heat-shock protein 70 (Hsp70) and glutathione peroxidase-1 (Gpx-1), were expressed in an oscillating manner in naïve rats (Figure S1B).

**Figure 1:**
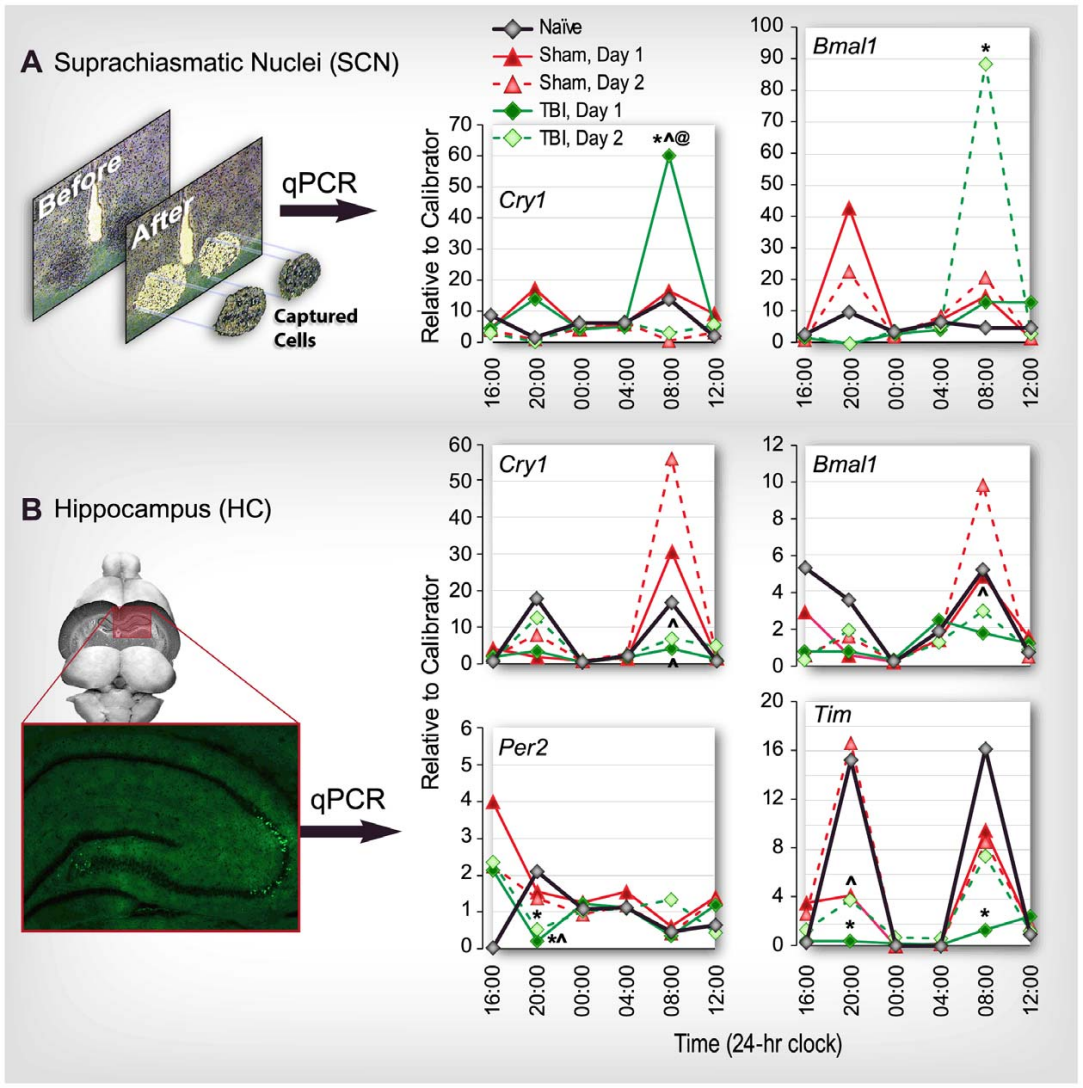
TBI alters cyclic expression of circadian clock genes measured in rat suprachiasmatic nuclei (SCN; A) and hippocampus (HC; B) using quantitative real-time PCR (qPCR). Rats received moderate fluid-percussion traumatic brain injury (TBI) or sham injury at 12∶00 or were naïve. Tissue was collected at 4 h intervals for 48 h after injury (n = 6 rats/group/time point) or starting at 16∶00 for 24 h in the case of naïve rats. Data for TBI and sham-injured rats for day 1 and day 2 are superimposed to emphasize diurnal variations in comparison with naïve rats over a 24 h period. **A:** SCN tissue was obtained by laser capture microdissection. Significant differences in mRNA expression were observed for *Cry1* at 08∶00 (20 h post injury) on day 1 and *Bmal1* at 08∶00 (44 h post injury) on day 2 (*upper right*). **B:** In whole hippocampal tissue, significant differences in mRNA expression were observed for four genes: *Cry 1* at 08∶00 on day 1 (20 h post injury) and 08∶00 on day 2 (44 h post injury); *Bmal1l* at 08∶00 on day 2 (44 h post injury); *Per2* on day 1 at 20∶00 (8 h post injury); and *Tim* at 20∶00 (8 h post injury) on day 1, 08∶00 (20 h post injury) and at 20∶00 on day 2 (32 h post injury). *p<0.05 vs. naïve same time; ∧*p*<0.05 vs. sham same time, same day; @*p*<0.05 vs. TBI same time, day 2. Standard error is not shown for clarity of presentation.

We found that TBI disrupted the expression of several of the circadian genes that were expressed in an oscillating manner. In neurons of the SCN, obtained by LCM (Figure 1A, upper left panel), a significant increase in the expression of *Cry1* was detected at 08∶00 (20 h post injury) on day 1 (TBI vs. naïve and sham at the same time point; *p*<0.05, as well as between TBI on day 1 vs. TBI on day 2 at the same time point; *p*<0.05). *Bmal1* expression was significantly increased on day 2 at 08∶00 h (44 h post injury; TBI vs. naïve and sham at the same time point; *p*<0.05) (Figure 1A).

In whole tissue samples of the hippocampus, expression of four clock-related genes was altered by TBI (Figure 1B). A significant reduction was observed in the expression of *Cry1* at 08∶00 (20 h post injury) on day 1 (TBI vs. sham at the same time point; *p*<0.05), and at 08∶00 on day 2 (44 h post injury; TBI vs. sham at the same time point; *p*<0.05).

Expression of *Bmal1* was significantly reduced in the hippocampus at 08∶00 on day 2 (44 h post injury) in TBI vs. sham at the same time point (*p*<0.05). *Per2* expression in the hippocampus was also significantly reduced at 20∶00 (4 h post injury) on day 1 (TBI vs. naïve and sham at the same time point; *p*<0.05) and at 20∶00 (32 h post injury) on day 2 (TBI vs. naïve at the same time point; *p*<0.05). Finally, expression of *Tim* was significantly reduced at 20∶00 (8 h post injury) on day 1 in TBI vs. naïve and sham at the same time point (*p*<0.05), and at 08∶00 h (20 h post injury) in TBI vs. naïve, and at 20∶00 (32 h post injury) in TBI vs. sham at the same time point (*p*<0.05; Figure 1B, lower panel). Two other genes associated with circadian function, the transcriptional repressor, Rev-erb alpha (also known as nuclear receptor subfamily 1, group D, member 1; *Nr1d1*), and the transcriptional co-activator, peroxisome proliferator-activated receptor-γ coactivator 1α (*PGC-1*α), also showed diurnal rhythms of expression in the hippocampus of naïve rats, but the expression of *Nr1d1* and *PGC-1*α was not altered by TBI (data not shown).

### TBI Disrupts Diurnal Expression of BDNF

**Figure 2:**
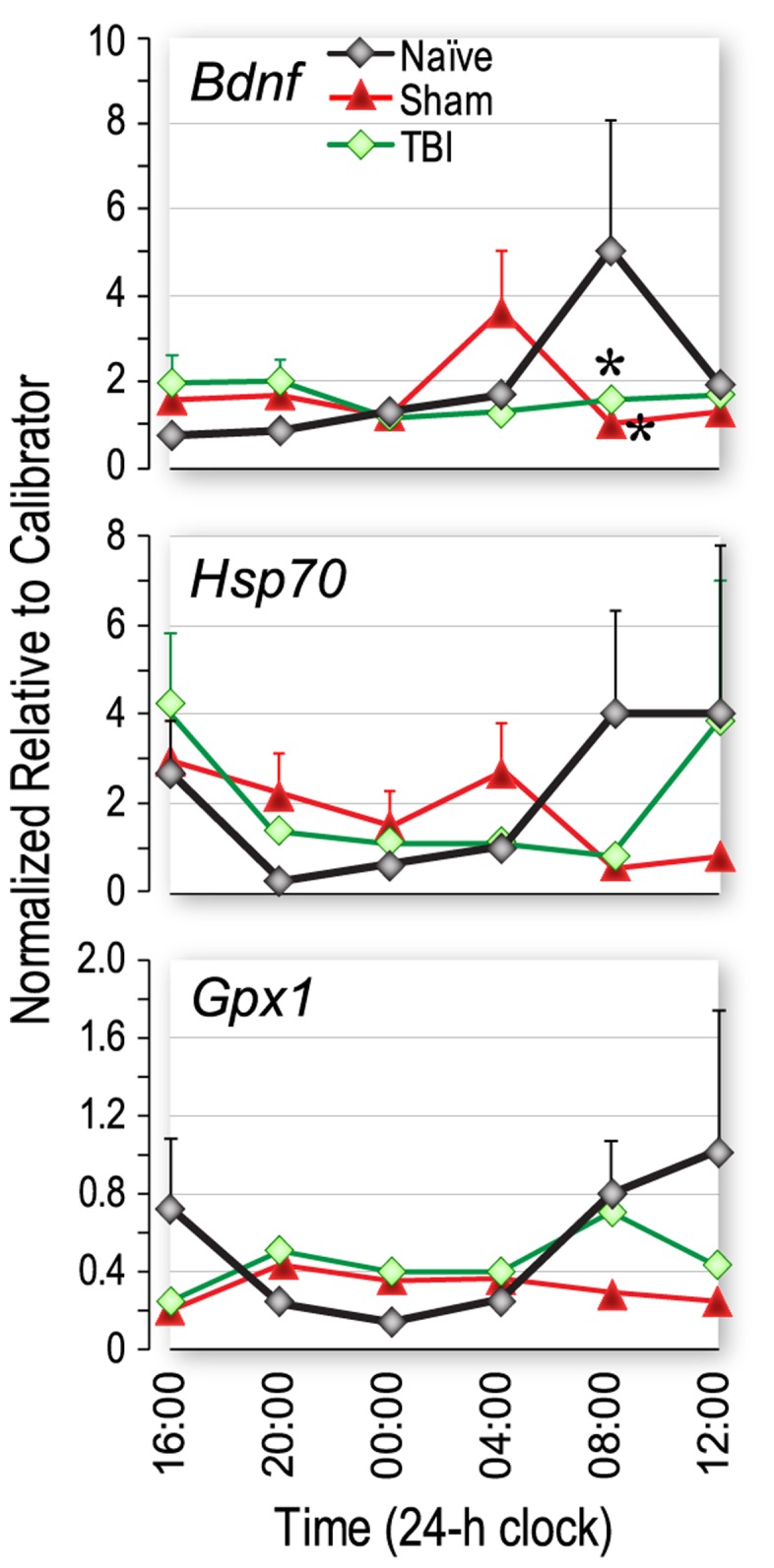
Injury-associated genes in the hippocampus that also demonstrate diurnal variations in mRNA levels. Traumatic brain injury (TBI) significantly altered the expression of brain-derived neurotrophic factor (BDNF), and appeared to alter the expression of heat shock protein 70 (Hsp70) and glutathione peroxidase 1 (Gpx-1) compared with naive rats and rats with sham injury. Rats received moderate fluid-percussion TBI, or sham injury at 12∶00 or were naïve. Tissue was collected at 4 h intervals for 24 h after injury (n = 7–8 rats/group/time point) or starting at 16∶00 for 24 h in the case of naïve rats. A significant reduction in mRNA levels of BDNF was observed at 08∶00 (20 h after injury) in both TBI and sham injured groups. *p<0.05 vs naïve, same time.

To determine whether disruption of circadian gene expression patterns in the SCN impacted clock-regulated genes in the hippocampus, we measured mRNA levels of BDNF, *Hsp70* and *Gpx-1* in hippocampal tissue from injured, sham-injured and naïve rats over a 24 h period. Expression of BDNF at 08∶00 (20 h after injury) was significantly reduced in the TBI group compared with the naïve group (*p*<0.05; Figure 2A, upper panel). Although not statistically significant, BDNF mRNA levels showed a tendency to be increased in the sham-injured group compared with the naïve group at 04∶00 h (16 h after injury). Large variability in *Hsp70* and *Gpx-1* data prevented these results from reaching significance (Figure 2B, C).

### Protein Expression Confirmation of Q-PCR Results

**Figure 3:**
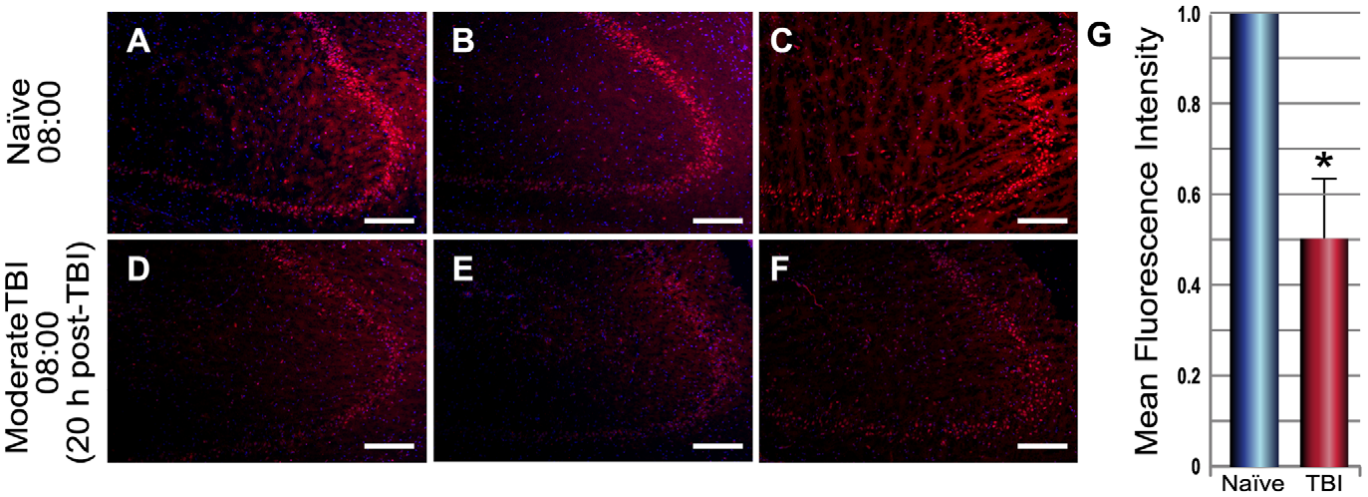
Immunofluorescence detection of Cryptochrome1 (CRY1) protein expression in hippocampal neurons from naïve rats (A–C) and injured rats (D–F). Compared with naïve, CRY1 protein levels were significantly reduced in the hippocampus after severe traumatic brain injury (TBI) at 08∶00 (20 h post injury; **D–F**). Mean fluorescence values were calculated for each image and corrected for the background using ImageJ and fold change was determined for TBI (n = 3) vs. naïve (n = 3). *p<0.01 vs. naïve (**G**).

Immunofluorescence and Western blot analysis were used to correlate the results of the circadian gene expression studies with protein expression. Based on the q-PCR results, three genes of interest (*Bmal1*, *Cry1* and *Per2*) were selected and hippocampal tissue was tested using immunofluorescence techniques for sensitivity to detect the protein product of each gene. We were able to detect PER2, CRY1 and BMAL1 in the dentate gyrus (DG) and PER2 and CRY1 in the *cornu ammonis* (CA; Figure S2). However, we found that PER2 and BMAL1 protein expression did not correlate with mRNA expression at the time points in which gene expression differences were detected. Differences in expression of CRY1 were apparent. Thus, we compared hippocampal tissue from injured rats with that of naïve rats at one time point 08∶00 (20 h after injury). CRY1 protein expression in hippocampal pyramidal neurons was reduced after injury compared with the naïve brain (Figure 3) and this result correlated with changes in *Cry1* gene expression at the same time point (20 hours) after TBI (Figure 1B). Using immunofluorescence, no other correlations between mRNA levels and protein levels were detected. This is most likely due to variability in a time lag between mRNA and protein expression. However, using Western blot analysis, we did observe a correlation between gene and protein expression of PER2 at 08∶00 (20 h post injury) and 20∶00 (32 h post injury; Figure S3).

### TBI Effects on Diurnal Rhythms of Locomotor Activity

**Figure 4:**
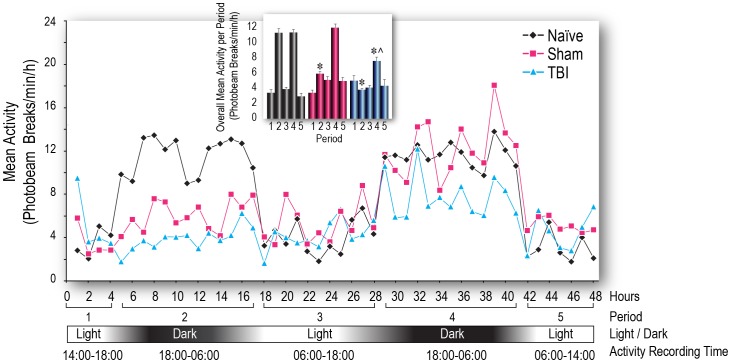
Time-course of locomotor activity in rats, measured over a 48 h period, following surgery. Rats received traumatic brain injury (TBI) or sham injury or were naïve and were recorded over an equivalent duration. Activity was counted as the number of beam breaks during 30 min intervals. The recording session was divided into 5 periods and the mean activity for each period was calculated for comparison. **Inset:** For the sham injury group, dark phase increases in activity were significantly inhibited during the second period (1^st^ dark phase after recovery from anesthesia), but recovered to normal levels by the fourth period (2^nd^ dark phase after recovery from anesthesia). However, in the TBI group, dark phase activity was significantly reduced during both the 2^nd^ and 4^th^ periods after injury. The mean activity counts per minute per hour is calculated as (the sum of two 30-min intervals [each hour])/60. *p<0.05 vs. naïve, same period; ∧*p*<0.05 vs. sham, same period.

In separate groups of rats, locomotor activity was monitored for 48 h after TBI or sham injury, or the equivalent time period for naïve rats. This was averaged over 30 min intervals to demonstrate a diurnal pattern of activity (Figure 4). Activity profiles were constructed by dividing the 48 h time course into 5 periods based on the light/dark cycle and the mean activity was calculated for each period (Figure 4, inset). Dark phase increases in activity were significantly reduced during Period 2 (the first dark phase measured) for both TBI and sham-injured groups compared with naïve rats (*p<0.05*). By Period 4 (the second dark phase), the sham group had returned to the same level as naïve However, the TBI group continued to show significantly reduced activity compared with both naïve and sham (*p<0.05*). Patterns of behavioral activity in the TBI and sham groups correlated with and mirrored the patterns of *Cry1* and *Bmal1* gene expression in the hippocampus (compare Figures 1 and 4) and like the gene expression data, were not phase-shifted.

## Discussion

This is the first demonstration that TBI alters circadian clock gene expression in the SCN and the hippocampus. TBI enhanced expression of *Cry1* mRNA at 20 h after injury and *Bmal1* mRNA at 44 h after injury in the SCN while reducing expression of these two genes in the hippocampus (*Cry1* mRNA expression is reduced at both 20 h and 44 h after injury and *Bmal1* mRNA expression is reduced at 44 h after injury). This is consistent with the concept of a feedback loop regulating oscillatory gene expression between the SCN and the hippocampus. These injury-induced alterations in expression of *Cry1* and *Bmal1* in both the SCN and hippocampus imply that TBI disrupts the timing of the central master clock. This is the first report that shows TBI disrupts the oscillatory expression pattern of several circadian clock and clock-associated genes in the SCN and the hippocampus, and for some genes (e.g., *Cry1* and *Per2*), these changes in patterns of mRNA expression correlate with changes in levels of protein expression and patterns of locomotor activity. Consistent with previous reports, our results showing diurnal patterns of expression of circadian clock-and clock-associated genes as well as injury-associated genes in the hippocampus [36] and SCN [37] are substantiated by the presence of protein products for these clock-genes in the hippocampus (CRY1, PER2 and BMAL1 in the dentate gyrus, and CRY1 and PER2 in the *cornu ammonis*).

Our primary finding that the expression patterns of key central-regulator genes, *Bmal1* and *Cry1* are altered by TBI in both the SCN and the hippocampus suggests that the communication between these two brain areas may be disrupted or dysregulated due to injury. Since the hippocampus mediates learning, memory and cognition, and diurnal regulation by the SCN is essential for proper hippocampal function, disruption of the oscillatory gene expression patterns in these two brain areas seems likely to play a role in the long-term cognitive effects of TBI.

The changes in gene expression observed here in genes with diurnal expression patterns are not phase-shifted; it is the magnitude of expression of clock genes that has increased or decreased compared with naïve and sham-injured animals at specific time points (this is true for the locomotor activity changes as well). Most of these differences occurred at 08∶00 or 20∶00 – at times when normal peak expression would occur. These points in time each coincided with 2 h after a change in phase (dark to light at 06∶00 and light to dark at 18∶00), so it seems that the internal system of entrainment is awry and either not able to mount the appropriate response or over-responsive to the daily phase changes. Because the oscillatory expression of *Cry1* and *Bmal1* are essential for the core autoregulatory transcriptional-translational feedback loops that compose the mammalian clock [38], [39], these disruptive effects of TBI on expression of these genes in the SCN and hippocampus support the hypothesis that circadian rhythms are important in learning and memory and disruption of these rhythms may have debilitating consequences for hippocampal function [40]–[42]. Our data provide evidence for a plausible molecular mechanism for circadian clock dysfunction and memory problems associated with TBI.

The circadian clock is intimately involved in cellular functions [43]; disruption of circadian rhythms in all organisms is associated with reduced fitness and increased vulnerability to disease [2], [44]. Circadian oscillations in hippocampal MAPK and cAMP activity suggest that the persistence of long-term memories may depend on reactivation of the cAMP/MAPK/CREB transcriptional pathway in the hippocampus during the circadian cycle [20]. Phase-shifting has been shown to disrupt circadian oscillations in MAPK and cAMP signaling pathways, which play critical roles in hippocampal-dependent memory [23]. The pattern of oscillations of the injury-associated genes BDNF and *Hsp70* seemed phase-shifted in the sham-injured group. Although we did not directly test for phase-shifting, it would be expected due to effects of general anesthesia (isoflurane) [45]. However, in the injured rats, the expression of BDNF, and to a lesser extent, *Hsp70*, appeared to be reduced at 08∶00, which is consistent with the results for the clock-related genes, suggesting that the effects of injury have a temporal aspect. Expression of *Gpx1* appeared to be damped at 08∶00 to 12∶00 in both the sham and TBI groups, again pointing to the effects of anesthesia [45].

Given the disruptive effects of TBI on endogenous circadian gene expression, we note with interest that TBI patients who commonly suffer from disorders of the sleep-wake cycle and depression often receive interventions using such compounds as melatonin (an endogenous neuroendocrine hormone) and fluoxetine (a selective serotonin reuptake inhibitor [SSRI], antidepressant), that, in addition to their primary therapeutic effects, also manifest chronobiotic properties [46], [47]. Although the antidepressant effects of fluoxetine are primarily associated with promotion of synaptic plasticity and neurogenesis [48], [49], it is conceivable that these may also be attributable to its chronobiotic effects, i.e. fluoxetine has been shown to phase advance the circadian clock in the SCN [50].

Both melatonin and lithium (a mood-stabilizer), reduce neurodegeneration in animal models of TBI; these effects have been attributed, at least in part, to antioxidant properties [51]. However, effects on circadian function may also play a role. Endogenous melatonin secretion can be disrupted in TBI patients. Patients with severe brain trauma exhibited clearly disrupted patterns of melatonin secretion, whereas those with less severe trauma showed relatively intact diurnal rhythms [52], suggesting that exogenous supplementation with melatonin may improve circadian clock function and potentially improve outcome after injury. Melatonin has been shown to contribute to improvement of hippocampal-dependent cognition in immature rats after brain injury [51] and, positively modulate memory processing [53]. Lithium also influences the circadian clock and enhances hippocampal neurogenesis [54]. Lithium acts via inhibition of glycogen synthase kinase-3β which phosphorylates and stabilizes *Nr1d1,* which we found to be expressed in the hippocampus [55]–[57]. Thus, melatonin and lithium may contribute to recovery from brain injury through multiple mechanisms that sometimes overlap to re-establish circadian function and to provide neuroprotection through anti-oxidant effects [58].

The dearth of clinically effective therapy for TBI patients makes the prospect of a novel therapeutic strategy to re-establish normal circadian rhythms worth investigating. Another potential compound for re-setting the master clock is rosiglitazone, a peroxisome proliferator-activated receptor-γ (PPARγ) agonist currently in use to regulate blood sugar in type II diabetic patients. It has been shown to be neuroprotective in brain injury [59]. Rosiglitazone directly effects expression of *Bmal1* in blood vessels, and is important in regulating circadian rhythms in blood pressure and heart rate [60]. Considering that *Bmal1* is a key component of the molecular circadian clock, rosiglitazone is another compound that could potentially be used to re-set circadian rhythms.

DNA damage from TBI can result in apoptotic and necrotic cell death. Aberrant cell cycle activation after TBI is known to cause cell death. Cellular circadian clocks are functionally linked to the cell cycle. Thus, DNA damage can alter the circadian clock within cells [61], [62]. Dysfunctional circadian rhythms could thereby enhance TBI-induced neurodegeneration by contributing to DNA damage and increasing expression of genes involved in oxidative stress and inflammation. Furthermore, nucleotide excision repair activity, up-regulated after TBI in the mammalian brain, is highest in the afternoon and evening hours [63], further indicating that functional outcome after TBI has a temporal component that could be manipulated therapeutically.

The disruption of circadian rhythms after TBI may influence the physiological response to therapeutic interventions; dysregulation of diurnal patterns of expression of genes involved in drug absorption, distribution, metabolism and excretion has been associated with lack of efficacy in clinical trials. Basically, drugs can have different effects when given at different times of the day [64]. In TBI patients, dysregulation of circadian rhythms may be a confounding factor in assessing the timing of, or response to treatment.

Here, we present a plausible molecular mechanism for the disruption or disturbance of circadian rhythms after TBI. However, since we did not examine clock gene expression or behavior beyond the first 48 h after injury, our studies merely suggest a conceivable mechanism for disturbed circadian rhythms but do not constitute proof. The coincidence of locomotor changes with changes in clock gene expression is suggestive but not definitive and requires further study that is beyond the scope of this initial investigation.

Understanding the molecular mechanisms of dysregulated circadian rhythms after TBI provides insight into other neurological and mental disorders such as Alzheimer’s disease and depression for which TBI is a risk factor [65]–[68]. Disturbed circadian rhythms are thought to contribute to the pathophysiology of these disorders. Thus, we speculate that pharmacotherapies with beneficial chronobiotic effects that may be used to treat TBI patients may also prove efficacious in mitigating circadian rhythm disturbances in Alzheimer’s and depressed patients.

## Supporting Information

Figure S1 **Diurnal variations in expression of clock and clock-associated genes in the hippocampus of naïve rats.** **A:** circadian clock and clock-associated genes: **B:** injury-associated genes with oscillating expression patterns. mRNA levels were determined using quantitative real-time PCR (qPCR). Tissue was collected at 4 h intervals starting at 16∶00 for a 24 h period. Data are presented as mean+SEM (n = 6 rats/time point).
(TIF)

Figure S2 **Immunofluorescence detection of three circadian clock gene products in hippocampal tissue taken from naïve rats at 08∶00.** CRY1 and PER2 protein are detectable in the dentate gyrus (DG) and pyramidal cells of the hippocampal CA1-3 regions (CA). BMAL1 is only detectable in the DG. The antibodies used are as follows: rabbit anti-CRY1 (1∶50 dilution); rabbit anti-PER2 (1∶50 dilution); rabbit anti-BMAL1 (1∶250 dilution). All antibodies were purchased from Santa Cruz Biotechnology Inc. (Santa Cruz, CA). Nuclei are stained blue with DAPI. Magnification is 10×.
(TIF)

Figure S3 **Western blot analysis.** Period 2 (PER2) protein expression in rat hippocampus at 08∶00 (20 h post-injury) and 20∶00 (32 h post-injury) demonstrates changes in relative protein expression levels after injury. Data did not reach statistical significance but does correspond with qPCR data at the same time points. Rats received moderate fluid-percussion traumatic brain injury (TBI) or sham injury at 12∶00 or were naïve. Data was averaged for each group (n = 3), normalized to GAPDH and quantified using UN SCAN it software (Silk Scientific).
(TIF)

Table S1 Primer and probe sequences for clock genes.
(TIF)
