# Supplementary figures and images for: Traumatic Brain Injury-Induced Dysregulation of the Circadian Clock
Source: PLoS One. 2012 Oct 3;7(10):e46204. doi: 10.1371/journal.pone.0046204 (PMC3463592; doi:10.1371/journal.pone.0046204)

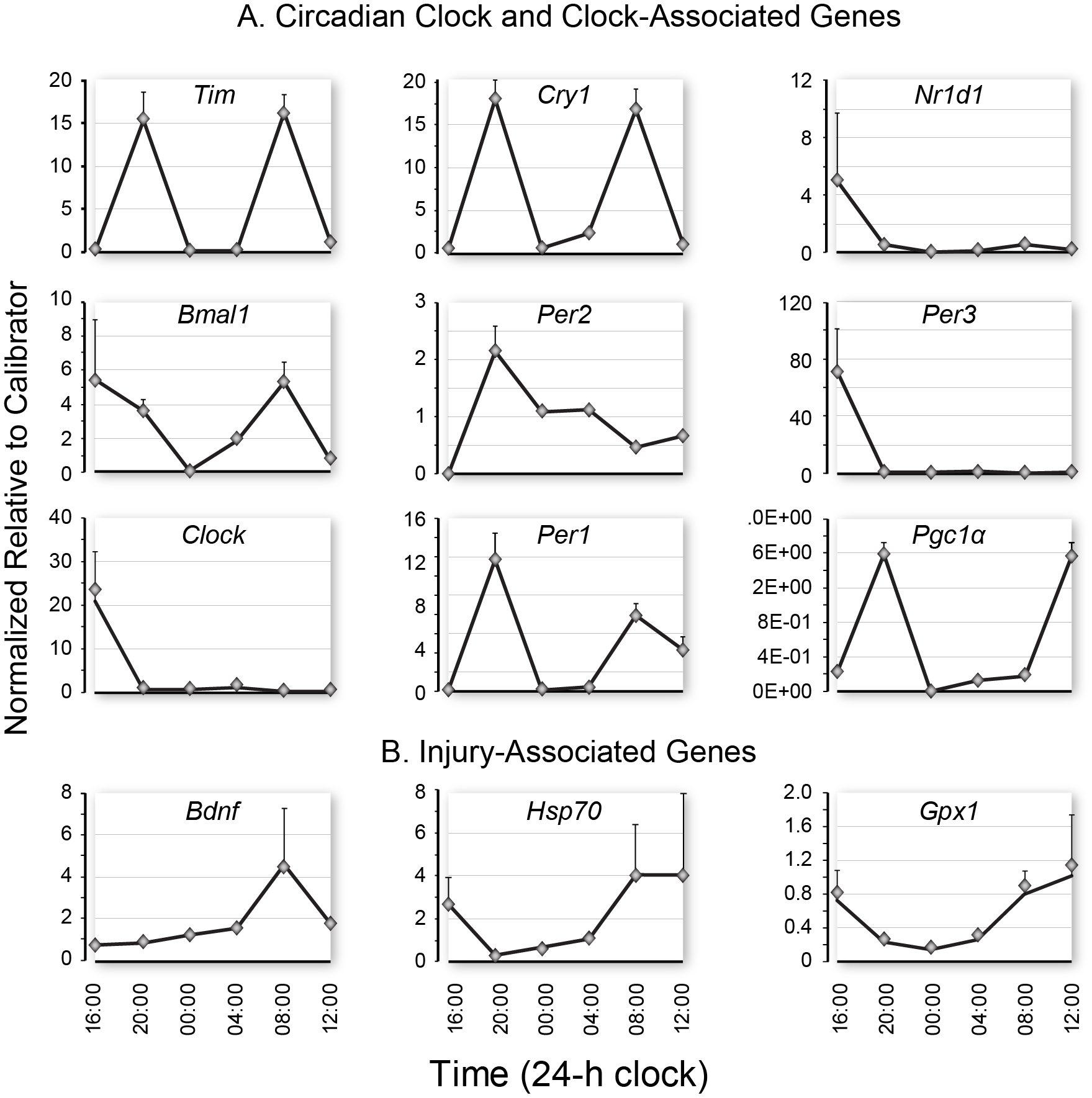

Supplement: Figure S1 — Diurnal variations in expression of clock and clock-associated genes in the hippocampus of naïve rats. A: circadian clock and clock-associated genes: B: injury-associated genes with oscillating expression patterns. mRNA levels were determined using quantitative real-time PCR (qPCR). Tissue was collected at 4 h intervals starting at 16∶00 for a 24 h period. Data are presented as mean+SEM (n = 6 rats/time point). (TIF) [file pone.0046204.s001.tif]

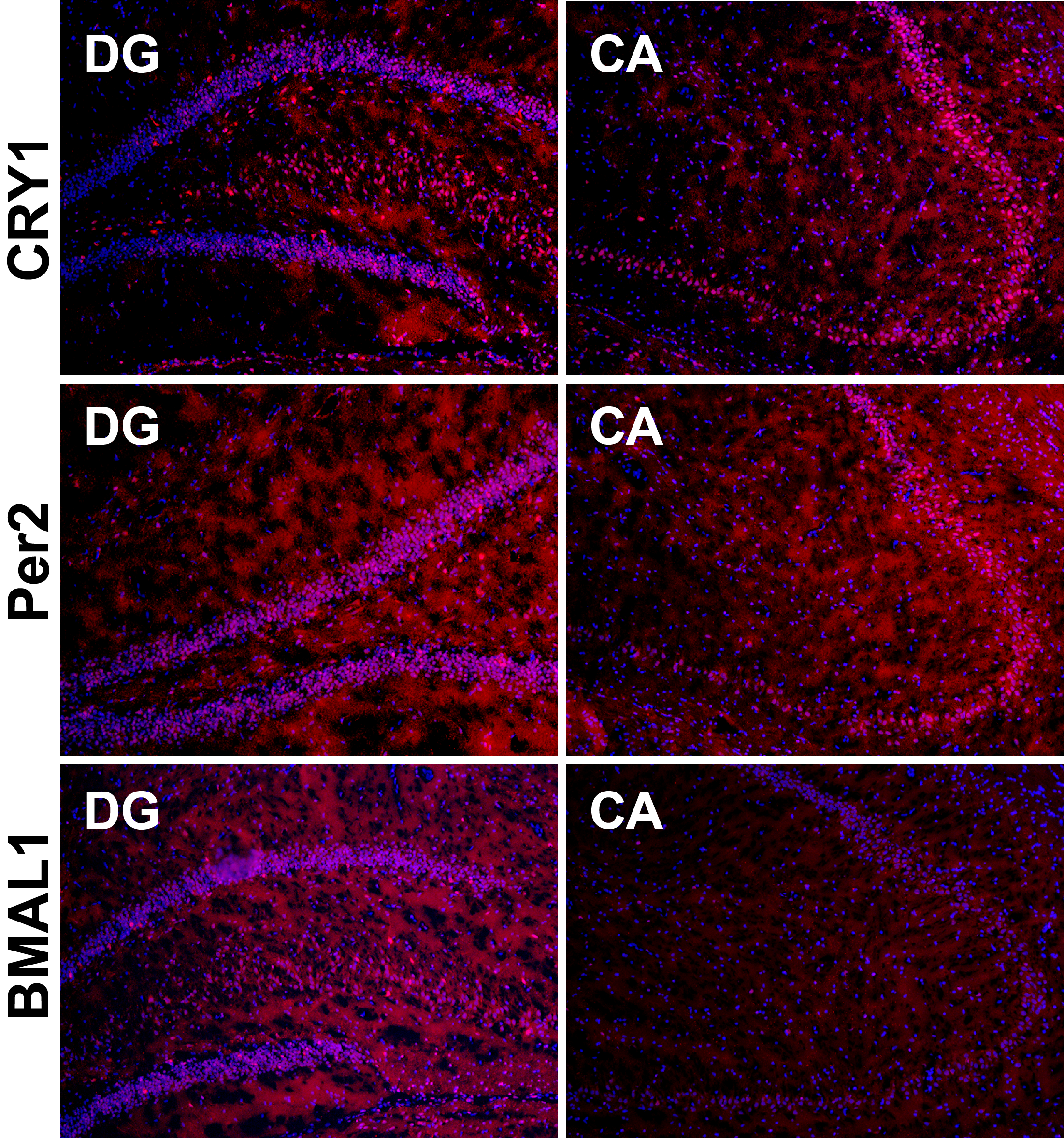

Supplement: Figure S2 — Immunofluorescence detection of three circadian clock gene products in hippocampal tissue taken from naïve rats at 08∶00. CRY1 and PER2 protein are detectable in the dentate gyrus (DG) and pyramidal cells of the hippocampal CA1-3 regions (CA). BMAL1 is only detectable in the DG. The antibodies used are as follows: rabbit anti-CRY1 (1∶50 dilution); rabbit anti-PER2 (1∶50 dilution); rabbit anti-BMAL1 (1∶250 dilution). All antibodies were purchased from Santa Cruz Biotechnology Inc. (Santa Cruz, CA). Nuclei are stained blue with DAPI. Magnification is 10×. (TIF) [file pone.0046204.s002.tif]

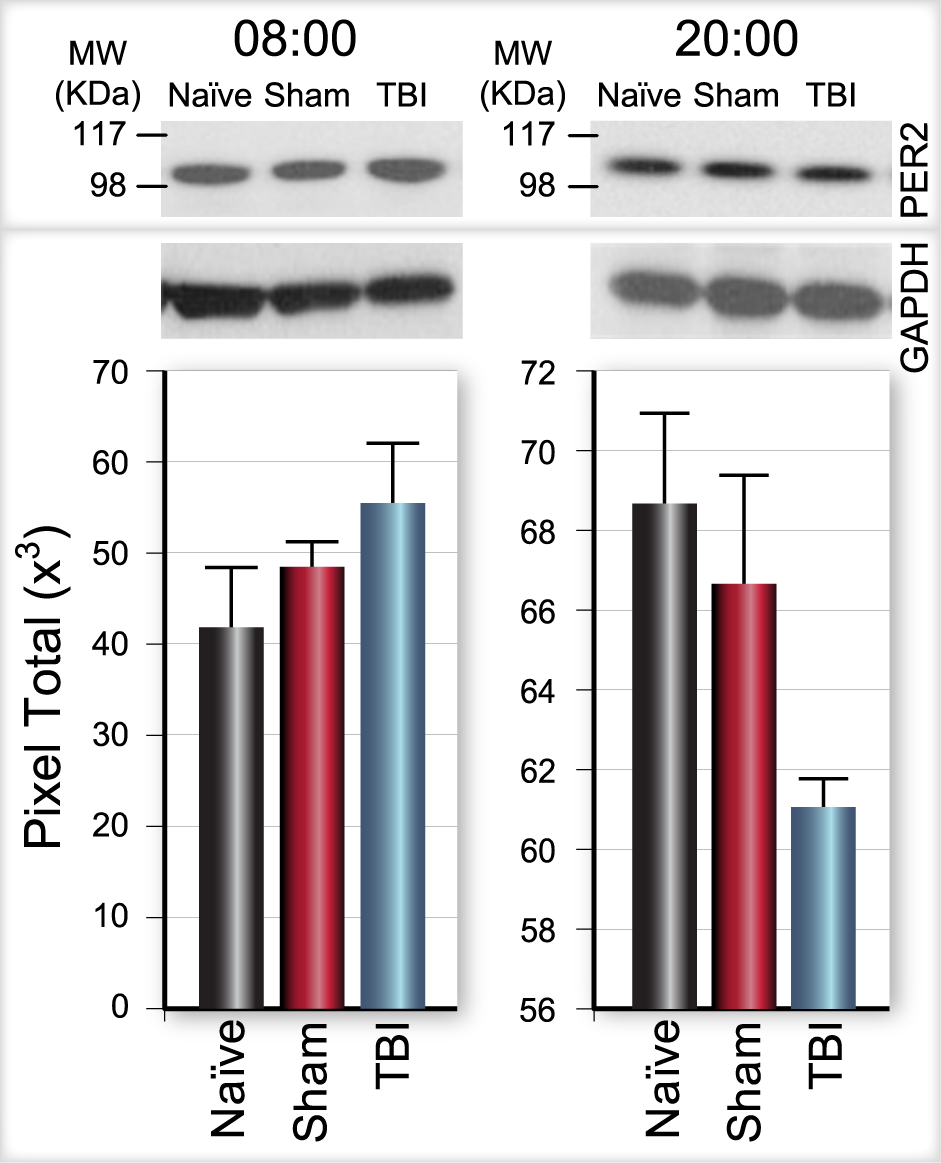

Supplement: Figure S3 — Western blot analysis. Period 2 (PER2) protein expression in rat hippocampus at 08∶00 (20 h post-injury) and 20∶00 (32 h post-injury) demonstrates changes in relative protein expression levels after injury. Data did not reach statistical significance but does correspond with qPCR data at the same time points. Rats received moderate fluid-percussion traumatic brain injury (TBI) or sham injury at 12∶00 or were naïve. Data was averaged for each group (n = 3), normalized to GAPDH and quantified using UN SCAN it software (Silk Scientific). (TIF) [file pone.0046204.s003.tif]

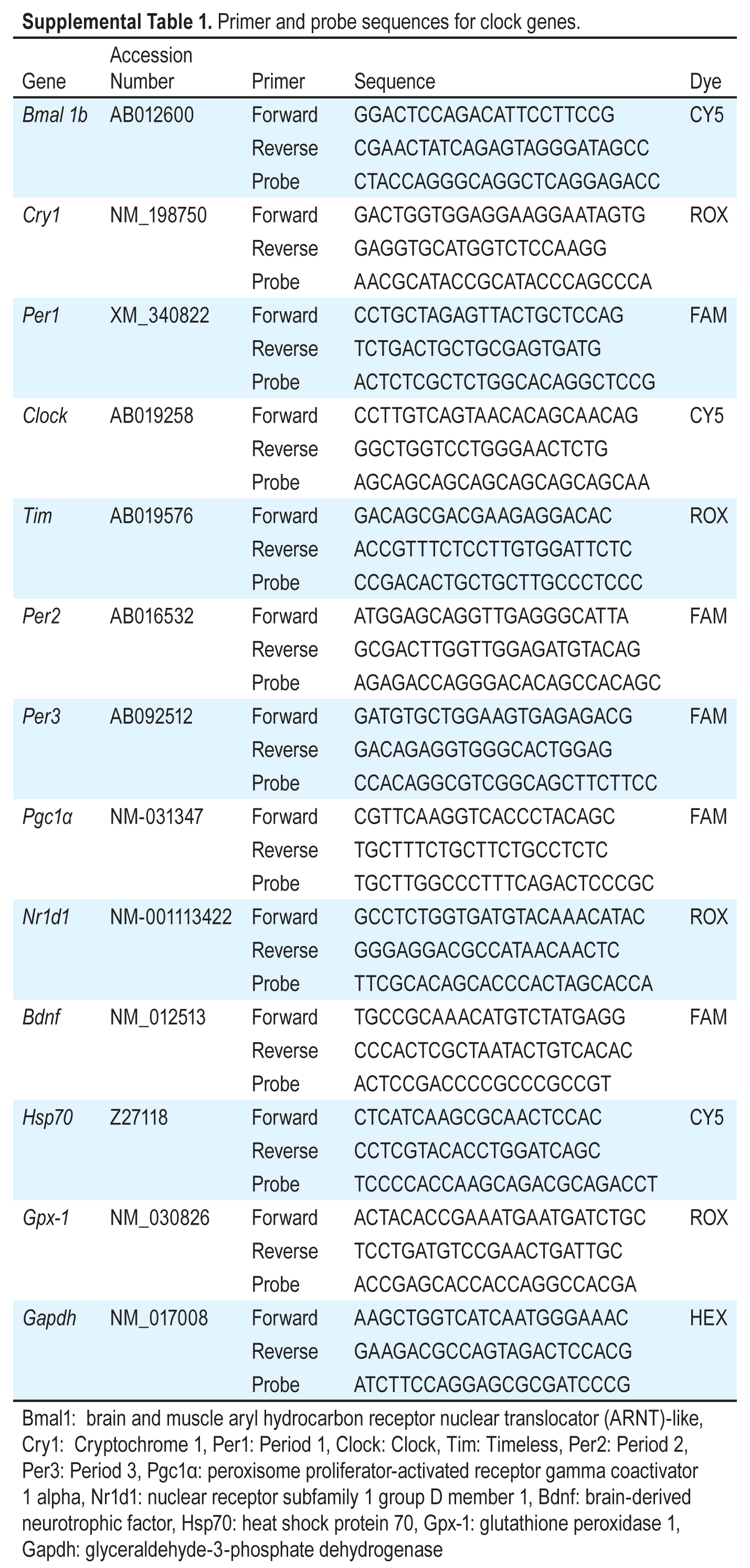

Supplement: Table S1 — Primer and probe sequences for clock genes. (TIF) [file pone.0046204.s004.tif]
